# Salmonella Osteomyelitis of the Proximal Tibia in a Previously Healthy Adolescent: A Case Report

**DOI:** 10.7759/cureus.5672

**Published:** 2019-09-16

**Authors:** Georgios Vynichakis, Michail Chandrinos, Stavros Angelis, Elefterios Bogris, John Ν Michelarakis

**Affiliations:** 1 Orthopaedics, General Hospital of Piraeus Tzaneio, Piraeus, GRC; 2 Ortopaedics, General Hospital of Piraeus Tzaneio, Piraeus, GRC; 3 Orthopaedics, General Hospital Hellenic Red Cross Korgialenio Benakio, Athens, GRC; 4 Orthopaedics, General Children’s Hospital “Panagiotis & Aglaia Kyriakou”, Athens, GRC

**Keywords:** salmonella, adolescent, osteomyelitis, child, tibia, blood culture bottle

## Abstract

Salmonella osteomyelitis is an uncommon pathological condition. Usually, it is associated with hemoglobinopathies or other underlying disorders. Osteomyelitis due to Salmonella is extremely rare in a previously healthy patient. We present a case of a 12-year-old previously healthy male who suffered Salmonella osteomyelitis of the proximal tibia as well as the diagnostic algorithm and therapy protocol. In our case, Salmonella osteomyelitis was identified by direct inoculation of the sample in blood culture bottles. Traditional cultures were negative. The practice of blood culture bottles seems to be essential for diagnosis, so the appropriate treatment is performed.

## Introduction

Osteomyelitis is uncommon in adolescents and children [1]. A wide range of bacteria could be the cause for osteomyelitis. The most common pathogen is Staphylococcus aureus counting for more than 50% of all cases. Streptococcus, Escherichia coli, Klebsiella and Proteus are other bacterial pathogens that can cause osteomyelitis [1-2].

Salmonella osteomyelitis is essentially scarce. It counts for 0.8% of all Salmonella infections and only 0.45% of all types of osteomyelitis [1-8].

Osteomyelitis due to Salmonella is extremely rare in healthy individuals. Only a few cases have been reported in the literature [1, 3-4]. Usually, Salmonella osteomyelitis is associated with hemoglobinopathies such as sickle-cell disease or thalassemia and it seems to be a significant cause of morbidity and mortality in these individuals [1, 3-8]. Other predisposing factors that have been associated with Salmonella osteomyelitis are the history of trauma or surgery, steroids, malignancy (lymphoma), liver and cardiovascular disease, autoimmune disease (systemic lupus erythematosus), diabetes, chronic granulomatous disease, immunodeficiencies and HIV infection [3-4].

## Case presentation

**Figure 1:**
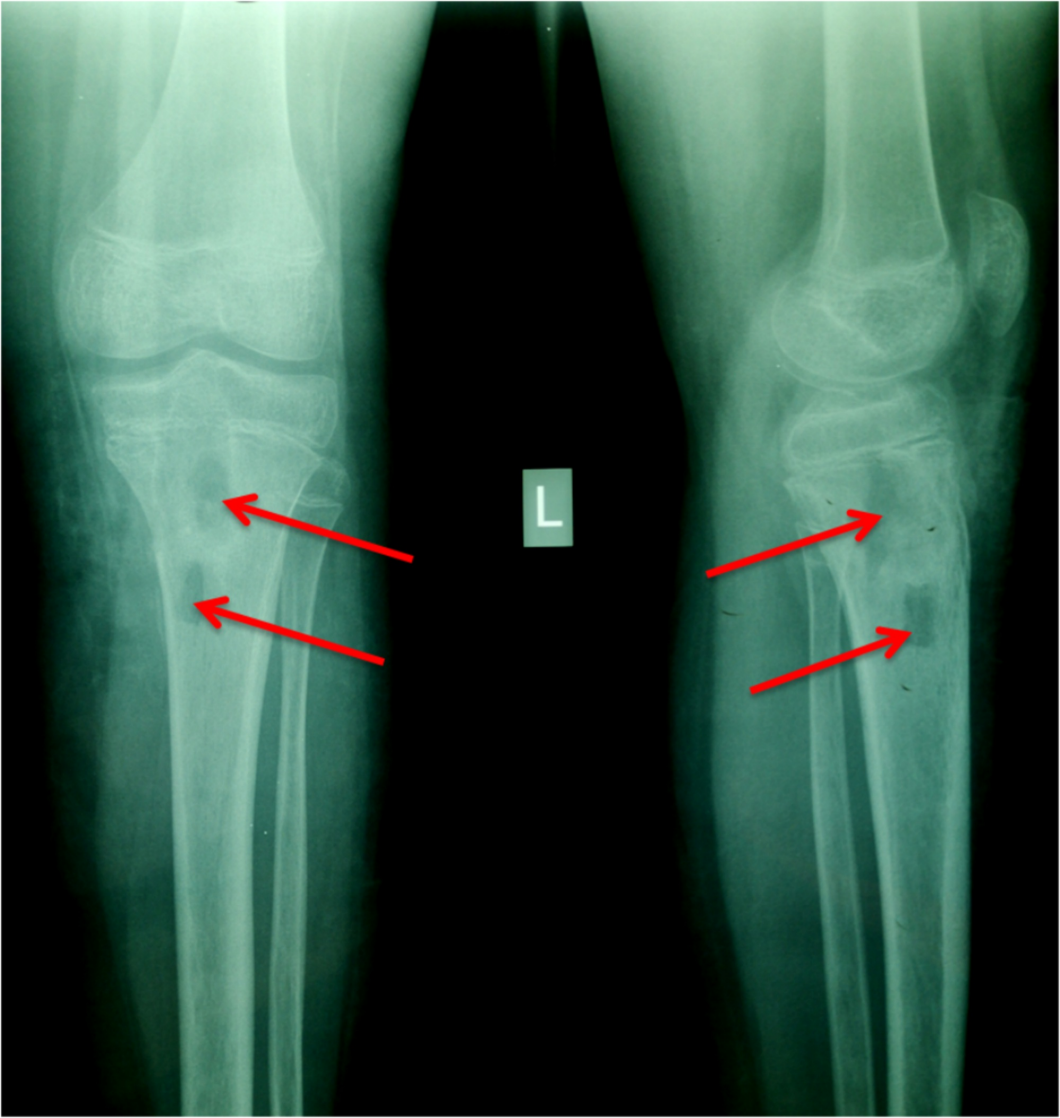
Knee radiograph: proximal tibia lytic lesions (red arrows)

A 12-year-old non-ambulatory male was referred to the emergency department of our hospital. He complained about pain in the left knee, fever (up to 40^o ^C), swelling, inability to move and walk properly. Regarding his personal medical history, no trauma or chronic disorder was referred by the patient or the parents. Clinical examination revealed swelling of the knee, stiffness, pain, and restriction of passive movement. Laboratory tests revealed a serum white blood cells (WBC) count of 12700/ μl (normal: <10000/μl), an erythrocyte sedimentation rate (ESR) of 49 mm per hour (normal: <25 mm/h) and C-reactive protein (CRP) 55 mg/L (normal: <5 mg/L) with normal liver function and electrolyte values. Blood cultures were negative. Radiographs revealed lytic lesions in the metaphyseal region of the proximal tibia (Figure 1). Osteomyelitis was suspected. The patient was admitted for monitoring, per os antibiotic therapy with amoxicillin and clavulanic acid and further evaluation.

**Figure 2:**
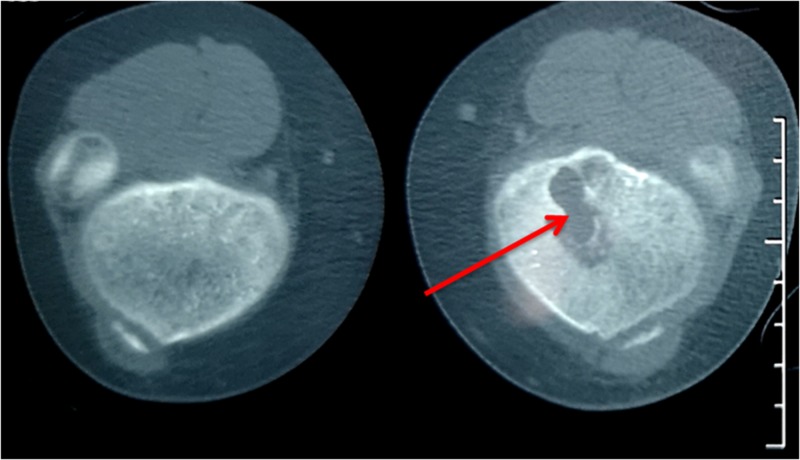
Knee CT: lytic lesion in the proximal left tibia (red arrow)

**Figure 3:**
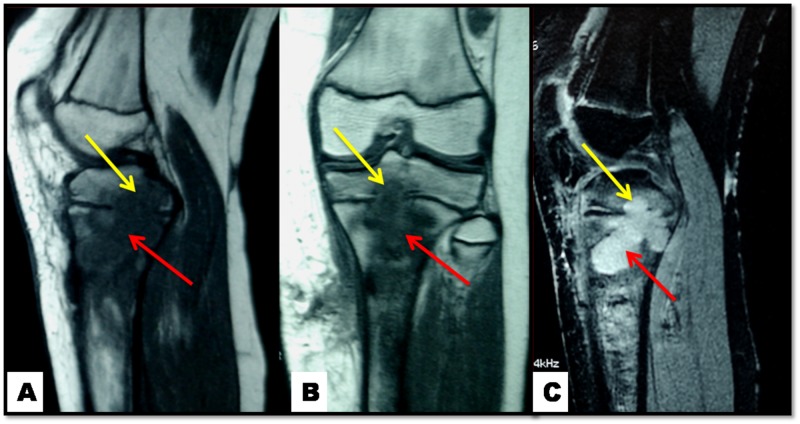
Knee MRI: lytic lesion at metaphyseal (red arrows) and epiphyseal (yellow arrows) regions A: sagittal in T1-weighted sequence; B: coronal in T1-weighted sequence; C: sagittal T2-weighted sequence

Lytic lesions were further evaluated with knee computed tomography (CT) and magnetic resonance imaging (MRI) (Figures 2, 3). The MRI and CT revealed that the lesions extended not only in the metaphyseal region but also in the epiphyseal.

After pediatric infectious diseases specialist's (PIDS) consultation a minimally invasive surgery was decided. Bone and lesion sample for cultures and histological examination was taken and curettage of the lytic lesion was performed. Cultures were negative but histological soft tissue examination revealed inflammation. Intravenous (IV) clindamycin was administered for 12 days. Per os antibiotic treatment (clindamycin) was totally administered for eight weeks.

**Figure 4:**
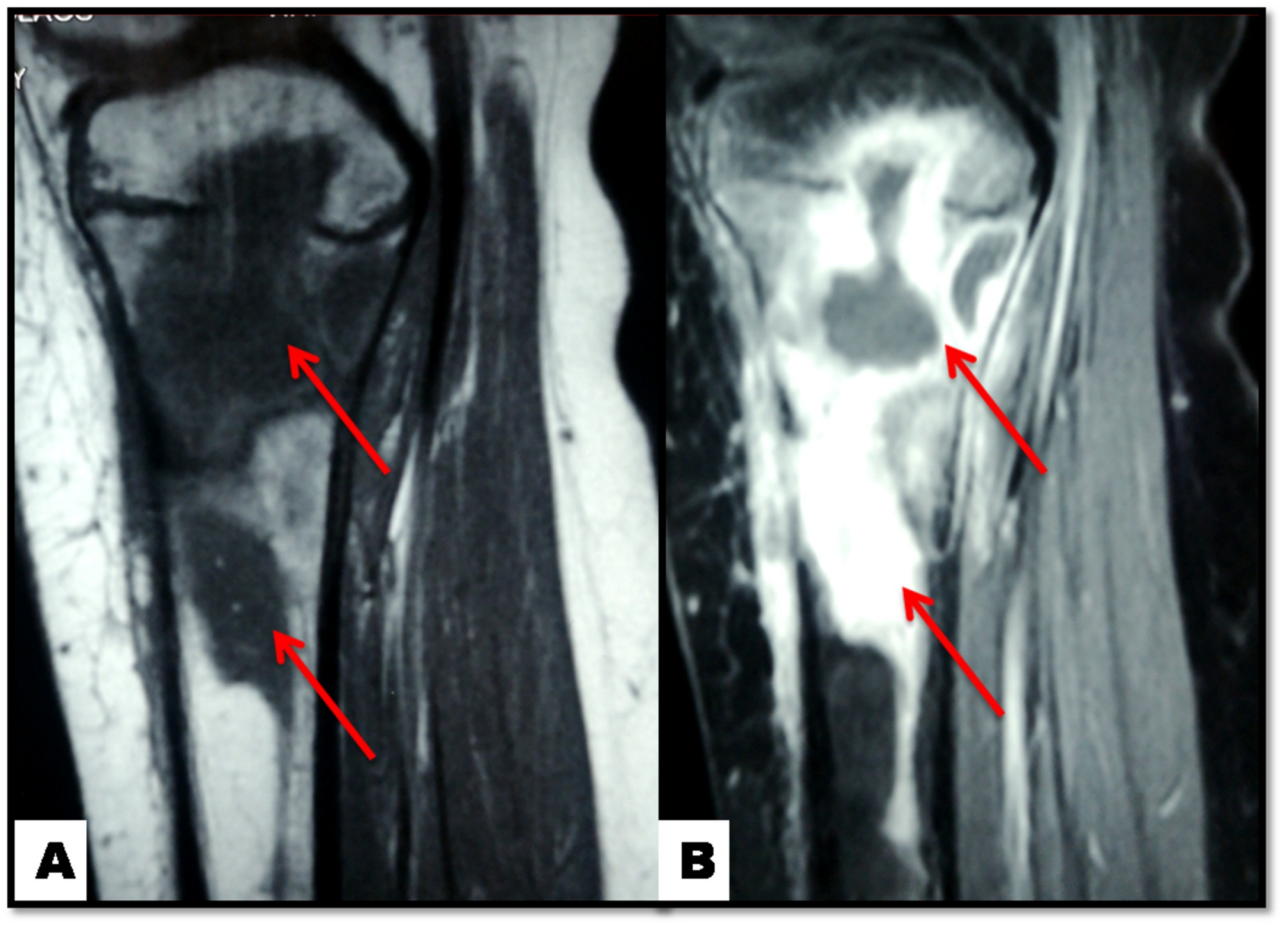
Knee MRI: osteomyelitis of the proximal tibia (red arrows) A: sagittal T1-weighted sequence; B: sagittal T2-weighted sequence

Three months post-surgery the patient returns experiencing relapse knee swelling and pain. The surgical incision was festering. Laboratory examination revealed WBC count of 8000/ μl, ESR of 28 mm per hour and CRP 4 mg/L with normal liver function and electrolyte values. The child was rehospitalized. After PIDS consultation, IV vancomycin, second knee-MRI and second surgery were decided (Figure 4).

Second surgery for curettage and debridement was performed. Samples for cultures were taken. Aspirates were cultured onto selective agar media and inoculated in blood culture bottles. Traditional cultures remained negative. The cultures inoculated in blood culture bottles revealed Salmonella non-typhi sensitive in cephalosporins and quinolones. After PIDS consultation, IV ceftriaxone was administered for 12 days and two weeks per os antibiotic treatment (ciprofloxacin) was followed as an outpatient.

**Figure 5:**
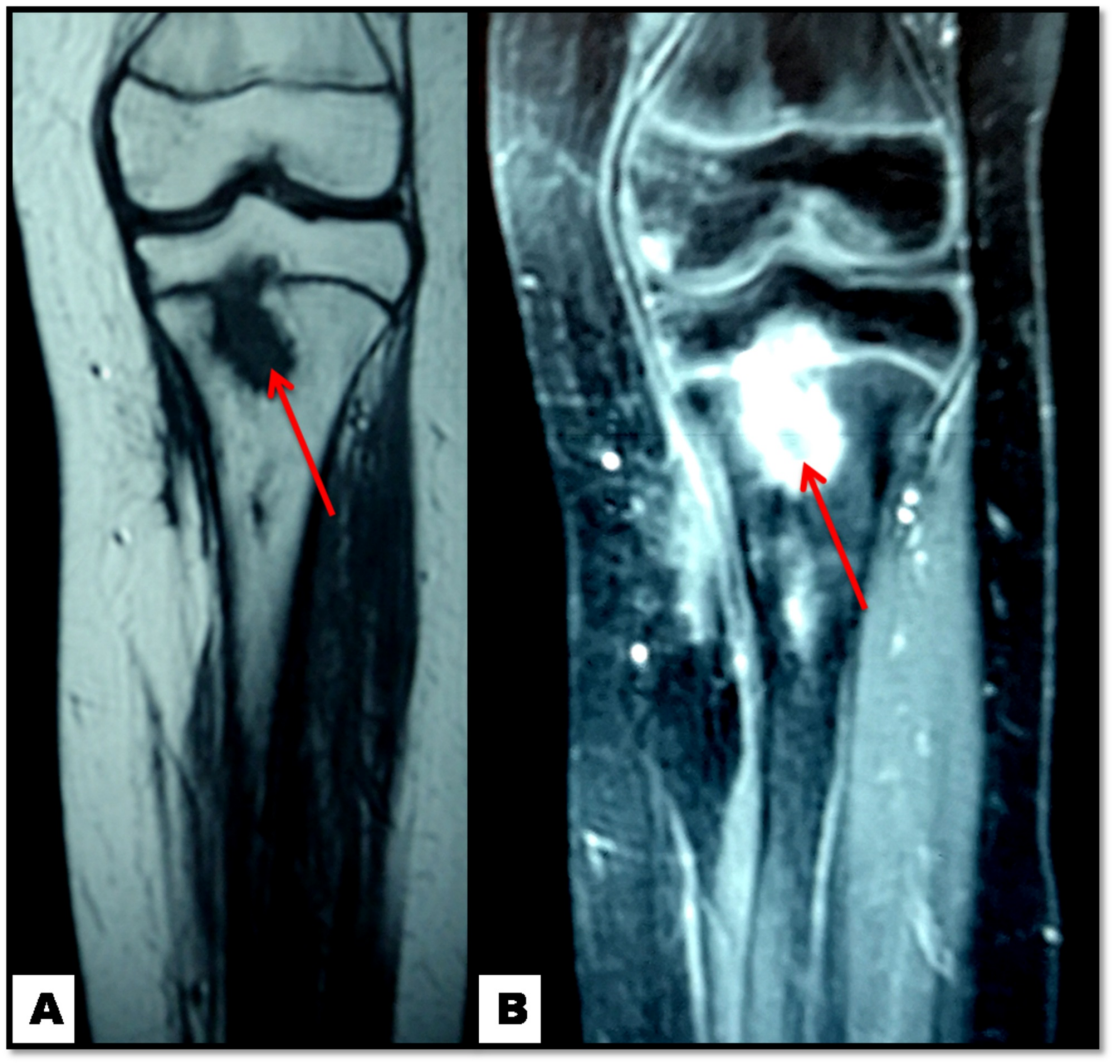
Knee MRI: osteomyelitis of the proximal tibia (red arrows) at six months follow up A: coronal T1-weighted sequence; B: coronal T2-weighted sequence

**Figure 6:**
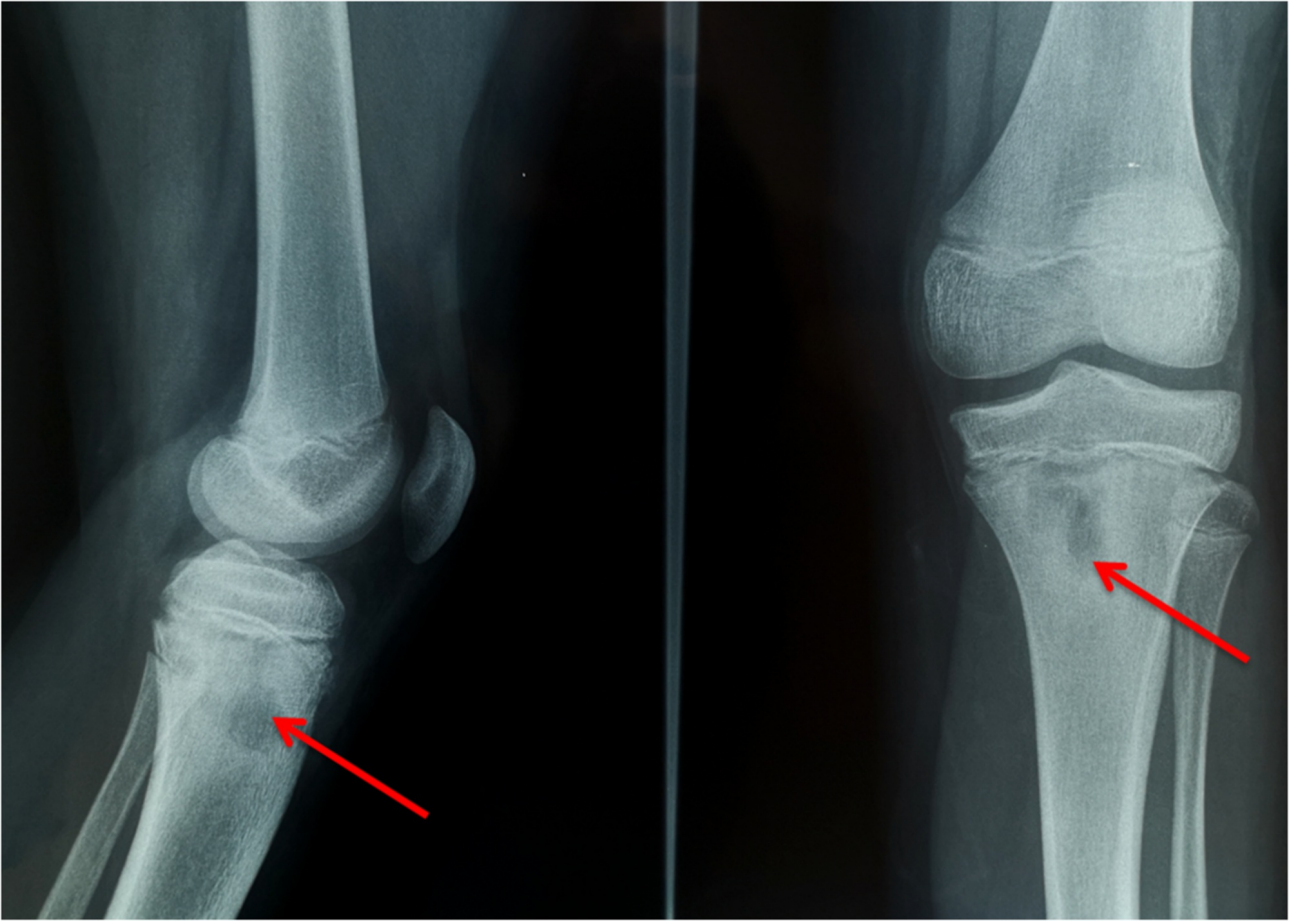
Knee radiographs at two years follow up: osteomyelitis of the proximal tibia (red arrows)

The patient was followed up by clinical, imaging and laboratory (WBC, ESR and CRP) examinations every week the first month, monthly the next five months and every six months until the two years follow up. At six month post-surgery follow up a knee MRI was performed (Figure 5). MRI revealed that the bone marrow had improved and the lytic lesion (Salmonella osteomyelitis) was reduced. At two years follow up the patient had fully recovered (Figure 6).

## Discussion

In humans, Salmonella infection can manifest with variable clinical symptoms. It can be divided into five syndromes: gastroenteritis (food poising), enteric fever (typhoid), bacteremia or septicemia, focal infection (including soft tissue or bone infection) and a chronic carrier state [1, 5].

Salmonella infection is one of the most common food-borne infections. It is caused by contaminated water, non-pasteurized milk and food (eggs, poultry) [1, 5-6]. Amphibians and cold-blood reptiles (like snakes, turtles and lizards) can cause Salmonella infections through skin contact (exotic pet reptile owners) or by use in folk medicine (snake-based medicines) [6].

Osteomyelitis is a known condition but a rare manifestation of Salmonella. Salmonella osteomyelitis is counting for 0.8% of all Salmonella infections and only 0.45% of all types of osteomyelitis [1-8]. The most common strains of Salmonella causing osteomyelitis in adults are Salmonella typhimurium, Salmonella enteritidis, Salmonella enterica subsp. arizonae, Salmonella typhi and Salmonella paratyphi [3-4]. Usually, Salmonella osteomyelitis is associated with hemoglobinopathies, such as sickle-cell disease or thalassemia and it seems to be a significant cause of morbidity and mortality in these individuals [1, 3-8]. In this population group, when a bone infection is presented, Salmonella osteomyelitis should be suspected [7].

Salmonella osteomyelitis is usually located in the diaphyseal region of long bones, typically in the femur and the humerus [3-4, 6]. Other bones commonly affected are the radius, ulna, tibia, and vertebrae [3]. Our case concerned the proximal tibia at the metaphyseal and epiphyseal region, which is uncommon. It was very difficult to diagnose because of the uncommon location of the lytic lesion and because our patient was a previously healthy child.

Clinical, radiological (with plain radiographs, CT scan, MRI scan) and laboratory evaluation should be provided in order to diagnose osteomyelitis. Salmonella osteomyelitis is difficult to diagnose. Clinical and radiological presents are indistinguishable from osteomyelitis caused by other microorganisms [1]. Signs of osteomyelitis caused by Salmonella are not revealed until 10 to 14 days after infection in plain radiographs and diagnosis is often delayed during the acute phase [1]. The duration of symptoms can range from a few months to several years [6]. In our case, the diagnosis of Salmonella osteomyelitis was delayed for three months.

Clinical deficits, radiographs, CT scan, MRI scan and inflammation markers are used to diagnose osteomyelitis. Blood cultures and cultures on agar media can identify the microorganism that causes osteomyelitis and the sensitivity in antibiotics, so the appropriate therapy is administered. In some cases though, it is difficult to identify the microorganisms in traditional cultures. In our case, Salmonella was isolated only when we inoculated sample in blood culture bottles.

Targeted therapy for Salmonella osteomyelitis, according to the results of the culture, is fundamental. Usually, Salmonella is sensitive to penicillin antibiotics, third-generation cephalosporin, fluoroquinolones and chloramphenicol [1, 3-8]. Salmonella infection requires extensive and sometimes multiple debridements in addition to prolonged antibiotic therapy [6]. Duration of treatment ranges from five weeks to six months [4]. In our case, Salmonella non-typhi was sensitive in all the above antibiotics. Our patient required two surgical debridement procedures and two periods of antibiotic therapy.

## Conclusions

The targeted treatment of Salmonella osteomyelitis is important and should be started as soon as possible. When traditional cultures are negative, inoculation of aspirates into blood culture bottles can be useful to diagnose Salmonella osteomyelitis in order to perform appropriate treatment.
